# Constituting link working through choice and care: An ethnographic account of front‐line social prescribing

**DOI:** 10.1111/1467-9566.13569

**Published:** 2022-10-25

**Authors:** Bethan Griffith, Tessa Pollard, Kate Gibson, Jayne Jeffries, Suzanne Moffatt

**Affiliations:** ^1^ Population Health Sciences Institute Faculty of Medical Sciences Newcastle University Newcastle Upon Tyne UK; ^2^ Department of Anthropology Durham University Durham UK; ^3^ Department of Social Work, Education and Community Wellbeing Northumbria University Newcastle‐Upon‐Tyne UK

**Keywords:** governmentality, link worker, logic of care, logic of choice, personalisation, social determinants of health, social impact bond, social prescribing

## Abstract

Link worker social prescribing has become a prominent part of NHS England’s personalisation agenda. However, approaches to social prescribing vary, with multiple discourses emerging about the potential of social prescribing and different interpretations of personalisation. The transformational promise of social prescribing is the subject of ongoing debate, whilst the factors that shape the nature of front‐line link working practices remain unclear. Based on 11 months of in‐depth ethnographic research with link workers delivering social prescribing, we show how link workers’ practices were shaped by the context of the intervention and how individual link workers navigated varied understandings of social prescribing. Following the work of Mol, we show how link workers drew differentially on the interacting logics of choice and care and trace a multiplicity in front‐line link working practices within a single intervention. However, over time, it appeared that a logic of choice was becoming increasingly dominant, making it harder to deliver practices that aligned with a logic of care. We conclude that interpreting personalisation through a logic of choice could potentially undermine link working practices that privilege care whilst obscuring the need for wider investment in health care systems and the social determinants of health.

## INTRODUCTION

Social prescribing involves the referral of patients from primary care to link workers who aim to offer personalised support and address non‐clinical concerns, usually by connecting individuals to community or third sector organisations (NHS England, 2019; The King’s Fund, 2020). In recent years, it has gained traction internationally (Zurynski et al., 2020) and is now a significant part of NHS England’s personalised care agenda (NHS England, 2019). Social prescribing link workers are increasingly operating within the NHS through newly formed Primary Care Networks^1^ that have been allocated NHS funds to either employ link workers directly, or indirectly through third sector organisations (NHS England, 2019). Some Primary Care Networks also separately commission supplementary social prescribing initiatives to meet specific local needs, whilst in some localities, schemes that pre‐date widespread NHS delivery are still in operation. With differential levels of collaboration between the various stakeholders, local landscapes continue to evolve (Husk et al., 2020; Tierney et al., 2020).

One result of this changing landscape is the range of link worker activities that now exist across England (Bertotti et al., 2019). Whilst some social prescribing interventions offer intensive and open‐ended support, responding iteratively to patients’ needs, others have well defined limits, lack flexibility and offer little more than signposting (Calderón‐Larrañaga et al., 2021). From relational to transactional (Calderón‐Larrañaga et al., 2021) or holistic to light touch (Kimberlee, 2015), the experiences of those delivering and receiving link working are variable (Gibson et al., 2021; Wildman et al., 2019). Alongside the more rewarding aspects of the role, link workers have described poor support, high staff turnover and emotional drain (NALW, 2020; Rhodes & Bell, 2021; The King’s Fund, 2022). The nature and availability of organisations available to link workers for onward referral has also become increasingly inconsistent, with significant changes, including reductions in availability, following the COVID‐19 pandemic (Morris et al., 2022).

### Tracing multiple discourses: What constitutes link working?

Given the range in social prescribing activity, it is perhaps unsurprising that multiple discourses have emerged about the potential of link working (Calderón‐Larrañaga et al., 2022). Often described as addressing ‘what matters to someone’ (NHS England, 2019, pp. 24–5) and promoted as a way to address health inequalities (Chng et al., 2021), social prescribing link working is also frequently embedded in discourses of supported self‐management, independence, behaviour change and reduced demand on both primary and secondary health care services (Calderón‐Larrañaga et al., 2022; Elston et al., 2019; NHS England, 2019). This has prompted scepticism about its transformational promise. Whilst some question the capacity of social prescribing to truly address the social determinants of health (Chng et al., 2021), others fear it could even exacerbate health inequality (Brown et al., 2021; Gibson et al., 2021). Perhaps in contrast to these understandings of social prescribing are those that promote a more person‐centred approach, where link workers attend to the differing needs of individuals through a focus on relationships within a network of care (Calderón‐Larrañaga et al., 2022).

Whilst these multiple and opposing discourses highlight the varied understandings of link working, its actual constitution remains elusive. Link working has been described on a spectrum (Calderón‐Larrañaga et al., 2021; Kimberlee, 2015), but what determines the front‐line nature of link working practices within this spectrum is less clear. In addition to the activities of individual link workers, existing literature points to the importance of training, workforce support, location and local ‘buy‐in’ in helping to embed link working (Hazeldine et al., 2021, p. 1849; Whitelaw et al., 2017). Although link worker freedom to develop ‘micro‐solutions’ is important (Hazeldine et al., 2021, p. 1850), it is also clear that the lack of buy‐in and wider support can limit the scope of link workers (Hazeldine et al., 2021; Mossabir, 2015). Different employment models and a lack of access to IT infrastructure add to the challenges of integrating link workers into Primary Care Networks, whilst pressure on other services, such as mental health services, can leave link workers dealing with complex caseloads for which they feel unprepared (The King’s Fund, 2022). Such findings resonate with the experiences of other front‐line health‐care workers, whose roles were shaped in nuanced ways by local contexts (Gale et al., 2018). For assistant physicians in the UK, routines became a balance between individual agency and structural permissiveness, whilst national policy also played an important part (Kessler & Spilsbury, 2019). To date, the impact of wider policy evolution on the nature of link working does not appear to have been explored in detail.

### Personalisation, choice and care

Despite the different discourses that exist about social prescribing, at a policy level, there is no doubt that it is now firmly embedded in the UK government’s personalised care agenda (NHS England, 2019). NHS England wants all staff within GP practices to have access to a link worker by 2022/23 (NHS England, 2019, p. 9), with claims that ‘social prescribing shares the values that underpin the wider personalisation movement in health and social care’ (Polley et al., 2017, p. 11).

Personalisation has become ubiquitous in UK health and social care policy but remains an ambiguous concept, with no fixed meaning (Needham, 2011). Such ambiguity can be useful in driving a political agenda but can make implementation challenging (Needham, 2011). In NHS policy, personalisation is a means of giving people the ‘choice and control’ that they have come to expect in other aspects of their lives (NHS England, 2019, p. 25). Translating a personalisation agenda that promotes choice into front‐line care can reveal tensions between its individualist assumptions (Needham & Glasby, 2014) and the complicated local contexts that can make choice appear no more than an illusion (Dalmer, 2019; Henwood et al., 2011; Miller & Barrie, 2020). In other settings, it has been suggested that a focus on choice can devalue care (Barnes, 2011) and obscure the need for wider investment (Needham, 2014). Indeed, concerns have already been raised that a focus on personalisation neglects the funding of community capacity needed for wide‐scale social prescribing to succeed (Morris et al., 2020).

Wider critiques of health interventions that promote choice often draw on Foucault’s concept of governmentality to show how people become responsible for managing their own health in response to wider social discourses (Foucault, 2008). In this context, it has been suggested that public health interventions frequently offer guidance to which reflexive individuals are expected to respond by *choosing* to govern their own behaviour (Petersen, 1997; Waring et al., 2016). Foucauldian perspectives thus draw attention to the potential discipline and surveillance associated with an emphasis on choice in the NHS personalisation agenda (Rhodes & Bell, 2021; Waring et al., 2016). Similarly, Mol (2008) considers the formation of subjects within health care, describing a prevalent ‘logic of choice’, which positions those being treated by health professionals as autonomous consumers, free to make, and responsible for making, their own decisions about their health. However, unlike Foucault, Mol also identifies a space for more ‘caring’ relations (Mol, 2008; Will, 2017). In contrast to a *logic of choice*, she advocates a *logic of care* that seeks ‘to act without seeking to control’ (Mol, 2008, p. 32) and recognises that individuals are embedded in socially meaningful lives. Care attends to uncertainty whilst ‘disentangle[ing] the practicalities’ (Mol, 2008, p. 60). Through a logic of care, personalisation becomes about relational understandings, negotiating and agreeing targets with close attention to individual context (Miller & Barrie, 2020; Mol, 2008). Individual experiences foreground the way in which ‘what matters’ is understood (Barnes, 2011).

By exploring these varied understandings of personalisation through choice and care, we can appreciate the challenges of developing front‐line practice, where ambiguity can give legitimacy to a range of practices (Needham, 2011). One might speculate that a focus on choice would align with social prescribing discourses that promote self‐activation and behaviour change, leaving little room for those that promote care, potentially favouring more transactional approaches to delivery (Calderón‐Larrañaga et al., 2021, 2022; Kimberlee, 2015; Mol, 2008). However, the opposing logics of choice and care almost certainly co‐exist (Mol, 2008), and it is the interplay of the two that becomes of interest when situating front‐line link working. To understand any type of *care*, one needs to observe it in practice (Brannelly, 2011). That is, we need to better understand what link workers do and the circumstances in which they do it.

## AIMS AND OBJECTIVES

By developing rich ethnographic accounts of link working within an established social prescribing intervention, we aimed to identify factors shaping delivery context and link worker practices through the interacting logics of choice and care, before examining how these practices resonated with contemporary social prescribing discourses and the stated aims and objectives of national policy.

## METHODS

This ethnographic research was conducted as part of the wider SPRING_NE study, a mixed methods evaluation of a large‐scale social prescribing intervention (Moffatt et al., 2022).

The intervention had been delivering link working since April 2015, following extensive pilot work. It allowed local primary care to refer individuals aged 40–74, with certain Long‐Term Conditions,^2^ to link workers who operated in an area of high socioeconomic deprivation. The service was commissioned by the local Clinical Commissioning Group (CCG) and part funded by a social investor through a Social Impact Bond (SIB). Re‐payments to the investor were made against the achievement of two primary outcomes: improved self‐management of Long‐Term Conditions and a reduced cost of secondary health‐care services. Link working was delivered by two third sector organisations that had been operating locally for some time. One of the providers had a longstanding history in community development, whilst the other was explicit about its commitment to behaviour change. Payments to these providers were not tied to outcomes but were linked to the number of beneficiaries they supported.

### Data collection

Face‐to‐face fieldwork occurred between August 2019 and February 2020. Due to the COVID‐19 pandemic, remote telephone methods were used between February 2020 and August 2020, when field work ended. From August 2019 to February 2020, Jayne Jeffries spent two days a week at the offices of link workers from the two provider organisations delivering the intervention. This included attendance at meetings as well as any scheduled training events. All link workers received participant information leaflets, and those agreeing to observation completed informed consent sheets. A total of 20 link workers agreed to face‐to‐face participation and returned a demographic questionnaire (16 female and 4 male). Between October 2019 and February 2020, Jayne Jeffries also shadowed a smaller number of these link workers (*n* = 8) as they went about their daily working routines. This included observing link workers meeting with clients at provider offices, GP surgeries or in the community and accompanying link workers when they were visiting clients in their homes. All clients observed interacting with link workers gave verbal consent. When possible, short informal interviews were conducted with link workers, during which they were asked to reflect on their practice. These were recorded in handwritten field notes that were subsequently digitised. Field notes also captured scenes encountered, pursuing meanings and reflecting on positionality.

During the period of face‐to‐face fieldwork, a further six formal interviews were conducted with link workers, and three focus groups were conducted across both provider organisations; with seven, six and three of the link workers, respectively. Seven of the eight link workers shadowed participated in the focus groups, but only two were formally interviewed, allowing us to capture a wide range of perspectives. Interviews used a semi‐structured interview guide and focus groups used a topic guide that focussed on eliciting link workers’ views about ‘what worked’ and how this varied between clients and link worker approaches. All participants were provided with information leaflets beforehand and signed a consent form prior to taking part. From February 2020, a further 13 telephone interviews were conducted with link worker managers and stakeholders. These were the focus of a separate analysis exploring the impact of COVID‐19 on the link workers and the intervention, which is presented elsewhere (Moffatt et al., 2022; Morris et al., 2022). All interviews and focus groups were audio recorded, transcribed and checked for accuracy by the research team. All data were anonymised with pseudonyms applied.

### Analysis

The final qualitative dataset comprised ethnographic field notes and the interview and focus group transcripts. Analysis was led by Bethan Griffith, who met regularly with Kate Gibson, Tessa Pollard and Suzanne Moffatt to review findings. A thematic content analysis was developed by coding all data and synthesising codes and categories into emerging themes of understanding. Interview narratives (‘what was said’) were compared with the observations (‘what was done’). Line‐by‐line coding was conducted using NVivo 12 (QSR International Ltd) with memos to assist in the process of moving from content‐based descriptive themes to more conceptual themes (Thorne, 2000). Following synthesis and theorisation of the data, new knowledge was re‐contextualised using wider literature.

### Ethics

Ethical approval was granted by Durham University Anthropology Department’s Research and Ethics Data Protection Committee.^3^ Regular meetings between the researchers also allowed for discussion of any emergent ethical considerations, paying close attention to their different positionalities.

## RESULTS

The SIB was seen as a pragmatic solution to local funding deficits, but ultimately meant that the intervention operated on a payment‐by‐results basis. It became clear during fieldwork that the funding model contributed to shaping the context in which link workers operated as well as the nature of link working delivered. We will begin by expanding on the context of delivery before examining link worker activities in further detail.

### The organisational context of link working

When new clients were referred to the intervention, they met with link workers and completed a standardised wellbeing assessment. This was used to set goals and was repeated regularly to monitor progress. Completion of sequential wellbeing assessments was one of the main routes by which the subcontracted providers secured payment, although this payment was not linked to any required improvement in wellbeing scores over time. In the early years of the intervention, completing assessments was less crucial because front‐loaded payments were provided as caseloads were being built. Later, during the period of our fieldwork, payments to providers could only be secured through completing assessments. This payment mechanism favoured a high volume of referrals as they increased the potential for wellbeing assessments, securing a steady stream of payment. Link workers were generally positive about using a wellbeing tool to structure client contact but felt pressured to complete them at the expense of other activities. It was clear that completing these wellbeing assessments was an organisational priority:

Staff frequently walk over to a white board in the corner of the office, marking off the [wellbeing assessments] they have completed with clients using a tallying system to indicate how many [assessments] (1, 2 or 3) they have completed for the month of August (or first, second, third). The initials of staff are written down the left hand side, the month and number of assessments across the top of the white board.

(Field Notes_07.08.19)

The pressure to secure referrals generated targets that were pre‐determined and fixed, aligning with a logic of choice that could be at odds with a logic of care, in which fixing a target before treatment is impossible (Mol, 2008, p. 54). This sometimes left link workers uncomfortable and conflicted as they operated at the intersection of choice and care. It could also cause confusion for clients, as Abby indicated:

So, I rang a lady who was supported by a previous colleague to introduce myself and get her in for a second [wellbeing assessment], but she was like, “Well, I don’t understand what you want from me. Why was I referred?” and I knew I was going to lose her, and I said, “Well, rather than you coming in, do you want to do the assessment over the phone?” and she did do it, and she was grateful for it afterwards. But it doesn’t sit comfortably working in that way. I know you don’t feel as if you think about the money, but that was one of the first things I was told when I started. So, [another link worker] had said to me, “We get X‐amount for a second [assessment],” and I thought that was commission. That was how it came across and I said, “Oh, I didn’t know that it was commission‐based,” and she said, “It’s not. It’s for the company.” and I was like, “Right.”

(Abby_Focus Group)

This focus on referrals and assessments shaped organisational priorities and made it harder for link workers to engage with complexity and offer the intensive support they felt some clients needed through privileging certain work routines over others. Abby went on to describe:

I thought, when I applied for the job, I’d be doing a lot more of that [support] than what we’re encouraged to do. So, my impression is that we’re kind of told to shy away from that as much as possible, because we’ve got so many targets for referrals and assessments. But in reality, I think it would be better if we were offering more one‐to‐one support into going out into the world, going to appointments, going to things.

(Abby_Focus Group)

This tension between meeting targets and the freedom to support clients resonates with the experiences of link workers in education (Fretwell, 2020) and of community pharmacists who were defined as much by policy as professional practice due to the ‘payments associated with contractual activity’ (Atkin et al., 2021, p. 339). Likewise, Sam described:

For me it’s incredibly frustrating to be based in practices where, if we were outside the [intervention] I would be busy from 6:00 am in the morning to 6:00 pm at night, very, very happily working with some people who need it…That imbalance is a result of structural strategic issues rather than patient need and is incredibly frustrating to me. I’ll leave it like that.

(Sam_Focus Group)

The impact the organisational focus on volume had on link worker routines was exacerbated by the differential engagement of local primary care. The original interventional logic dictated that primary care staff would refer eligible clients following the integration of link workers into GP practices. The reality was patchy primary care engagement that meant referral rates were low, as described elsewhere (Elston et al., 2019). Entrenched professional hierarchies were evident when link workers talked about their interactions with practice staff and their various attempts to increase the visibility of the role and encourage referrals. Ultimately, link workers increasingly had to secure their own referrals to meet targets. In some instances, they had to identify eligible clients from a list and telephone them to offer the intervention before asking a GP to complete a referral form. Differential access to practice IT systems and the persistence of paper referrals in some practices added to this arduous process. Sam spent all his time at one GP surgery generating referrals, not seeing any clients there. It became clear during one visit how hard it was for Sam to navigate relationships with staff at the surgery:

The link worker smiles and says hello to the staff as he picks up a small A5 book, opens it and begins to write our details inside… The staff do not respond to the link worker or acknowledge his presence with a nod or similar. A member of staff enters the reception office a few minutes before we leave, the link worker looks up, smiles and says hello to the staff member, who is perhaps a GP – he smiles at the link worker, but no hello…

(Sam_Field Notes)

After going upstairs to a shared office, Sam retrieved a folder with a list of eligible patients. He called a few to explain about the intervention and ask if they would like the GP to refer them. He later explained:

“last week I made 5 referrals and I am still waiting for a GP to sign those off” – Sam must submit a request to a GP seeking permission/consent to offer the [intervention] to a patient, [he] can call the patient to provide them with information regarding the [service], but he cannot book a “new client” in for an appointment until he receives this permission. The signing off is the GP signing the referral form and returning it to the link worker.

(Sam_Field Notes)

Unsurprisingly, the whole process was time‐consuming and left less time to spend with clients who were increasingly followed up over the telephone, rather than face‐to‐face contact. These routines were not consistent across all GP surgeries. Some link workers described better integration with primary care, having a designated room to see patients, and access to the practice IT system. Some link workers actively created opportunities to meet primary care teams by accessing communal spaces such as staff rooms. Link workers described how this required them to be ‘brave’, drawing attention to how the perceived hegemony of primary care shaped the context of link working routines alongside the organisational structures identified above.

Overall, the delivery context left link workers conflicted as individual subjectivities and organisational priorities became variably aligned and work routines were constrained in different ways. Whilst one provider had always been explicit about an approach that promoted empowerment and behaviour change, resonating with a logic of choice, there were examples, from both providers, of deeply relational link working practices that aligned with a logic of care. However, over time, routines that easily met organisational targets had become more common, and it had become harder to offer the undifferentiated support some link workers favoured. There appeared to have been a convergence in provider approaches towards behaviour change, suggesting organisational structures increasingly aligned with a logic of choice. Ultimately, some link workers felt unable to perform the role in the way they had imagined.

Whilst the organisational gaze became more focussed on outcomes, through a logic of choice, individual link workers tinkered with the role at the intersection of choice and care. The rationalities that shaped organisational structures were variably enacted and resisted on the front‐line, resulting in a spectrum of practices. We now turn the reader’s attention to exploring the nature of these practices in further detail.

### The content of link working

As stated, the aims of the intervention were to improve health outcomes and quality of life for those with long‐term conditions whilst also reducing health‐care costs. Recent job adverts seen from both provider organisations highlighted ‘empowerment’ and ‘behaviour change’, both terms that align with a logic of choice, as important mechanisms for achieving these aims, yet individual link workers interpreted the role in different ways. Link workers had a range of professional backgrounds, including roles in the community and voluntary sector as well as clinical roles within the health sector. Individual narratives about the role were often ideological but frequently conflicted, appearing to draw differentially on multiple social prescribing discourses (Calderón‐Larrañaga et al., 2022). Hilary, for example, had worked in health care for some time before becoming a link worker and acknowledged her familiarity and ease with working to targets, favouring a focus on individual behaviour change that aligned with the organisational gaze. In a focus group with colleagues, she explained:

The first thing is getting to know them to build a rapport. What I want them to get out of it is the courage to actually look after their own conditions, because for so long it’s you go to the doctor, the doctor says this, you come out, you’ve got your medicine, that’s fine and off you go and everybody’s happy. Or you go to [personal independent payment^4^] or [employment support allowance^5^], get your benefits and everyone is happy. That can’t go on because everyone has got to start taking control of their conditions.

(Hilary_Focus Group)

For Hilary, link working needed to be about more than *linking*, it needed to focus on behaviour change and empowering clients to ‘take control of their conditions’. Elsewhere, she was keen to distinguish between her own role and that of a support worker, contrasting her approach to that of a colleague who simply asked clients what they needed and assisted as possible, even helping one client to wash his dog. Using the newly created spaces of link working, Hilary drew on her historical experiences of bio‐medicine and its power frameworks to encourage individual responsibility and lifestyle change. Within this space, clients’ behaviour could be inspected and modified. This sense of intimate inspection was also apparent when link worker Abby visited one client in his home, and was intensified by the wellbeing assessment:

The client states that nothing has changed, [Abby] smiles, she continues by mentioning the [wellbeing assessment], stating that in order to say she has done her job properly she needs to complete one today. The client agrees… [Abby] asks what they eat, his wife states, “generally it is meat, potatoes, salad. But he gets his head stuck in the laptop and doesn’t get dressed, sometimes until the afternoon”… [Abby] returns to the client and asks him if he would agree with the comments made regarding his diet; he agrees. She asks him about exercise and he tells her, “I walk to the front of the gated community”. [Abby] asks, do you want to change? The client says “No”. [Abby asks] “How are your energy levels? In your notes it says that you often feel fatigued, have 6–8 hours sleep each night, and are a non‐smoker. Has that changed?” The client says, “It is about the same”. His wife interjects, “Last month you drank [a lot of alcohol].”

(Abby_Field Notes)

Such examples of inspection resonate with a logic of choice that focuses on an individual’s responsibility to change behaviours, in this case, diet, exercise, alcohol consumption and sleep. Such approaches often work by reinforcing the limits of acceptable behaviour and marginalising context (Foucault, 2008; Mattioni et al., 2021; Mol, 2008). However, in describing how link working was becoming increasingly about this approach, Hilary was also able to recognise how individual circumstances could make this challenging, despite her earlier criticism of colleagues attempting to offer unconditional support:

It is causing some people [other link workers] to be restless, because it’s much easier just to be a link worker. It really is. Just do an assessment, bang, bang, bang, link is there, there and there. That’s easy. That misses the person. That treats them more like a condition. You’ve got to see the person. The person is suffering with a bereavement, or there is so much they haven’t told you, and that’s what you’ve got to get behind.

(Hilary_Interview)

This introduces tensions and contradictions that were frequently evident and highlights the difficulties with discourses of empowerment and choice if people are not in a position to deploy them. In line with a logic of care, Hilary’s comments also draw attention to context (Mol, 2008).

Link worker Lucy, had her own experience of ill health and was keen to acknowledge the complexity of many clients’ circumstances. She had been instrumental in establishing a local community group that allowed different clients to meet and chat. She often had informal contact with clients, perhaps to remind them of a meeting at the job centre or to turn up to an appointment:

Actually, if I was a nurse and I had a client who was very anxious or lacking in confidence, I would think, “There’s no point sending them to a link worker if they’re just going to give them another phone number, because this person’s not going to access it.” I would send them to someone who was going to give them the time and take them, maybe, or sit with them to help them fill in the form because that’s an assessment you would make. I mean, I feel quite strongly about this, and I know a lot of people have a different idea and we’re not supposed to handhold.

(Lucy_Focus Group)

In contrast to responsibilising, Lucy’s approach might be thought of more as helping clients through a logic of care that allowed her to ‘disentangle the practicalities’ shaping engagement (Mol, 2008, p. 60). There is, however, a hesitancy in her response, suggesting her approach was not always aligned with the horizons of the intervention or indeed wider discourses that frame dependency as problematic (Calderón‐Larrañaga et al., 2022; Tierney et al., 2020). In short, Lucy, like Hilary, acknowledged the importance of context but diverged in her desire to offer person‐centred support. Abby illustrated the embodied nature of this style of support as she made an impromptu stop off at a different client’s house to deliver some paperwork ahead of a meeting that the client was due to attend at the benefits office:

The link worker recognises that the conversation is difficult for the client; she moves from the sofa to the floor, squatting down sitting on the floor in front of the client. This gesture indicates the friendship between the client and link worker. It makes the client feel comfortable; the link worker asks her again how she is feeling about it (the meeting). The link worker remains on the floor, looking up at the client, she continues to smile, asking if the client is okay. The client states that she is; she thinks so. The link worker tells her that she has brought a number of documents to support her case at her meeting; she takes four letters from her bag. The first shows the smoking cessation course the client attended, the second and third are two [intervention] letters, written by the link worker using the client’s case notes, the fourth, is a letter from Welfare Rights, showing the impact that her health has had on her finances and debt management. The client is happy with the letters from the link worker, who tells her that she didn’t want to post them to the client. She wanted to bring them to ensure she received them, and so she could check in on how the client was feeling before her meeting. The client states that she is grateful and appreciative of the extra work that the link worker has done for her. The link worker reassures her about the appointment, asking if she has any other questions and encouraging her to be in contact up until and following the appointment if she needs to talk again.

(Abby_Field Notes)

Abby pays close attention to any perceived power differentials in her gestures, working to provide an equal exchange that resonates with a logic of care. The nature of the support provided by Abby was echoed by other link workers’ descriptions of how ‘*just listening’* or ‘*being there*’ helped clients feel more confident, as Lucy suggested above, or taken more seriously. It illustrates how practices could be embodied and delivered within a logic of care that paid close attention to individual context and need. In practical terms, this meant addressing what mattered to the client, such as securing income, improving housing conditions or becoming more socially connected. Within this approach, a client’s ‘goal’ was not decided through a wellbeing tool but might be as simple as ‘*attending the next appointment*’. This desire to support clients frequently involved navigating and mitigating the structural barriers to health and wellbeing beyond individual control, aligning, once more, with a logic of care that recognised context and biographical disruptions (Mol, 2008).

Following their initial consultation with a client, link workers would suggest ways they might achieve their goals, which, under the model of link working, often meant onward referral or ‘linking’. Link workers were observed referring clients to exercise groups and cookery classes, amongst other activities, with certain referral pathways, such as local exercise classes, used more frequently. However, the intervention operated in an area of significant socioeconomic deprivation and, whilst the organisational gaze favoured promotion of individual choice, link workers felt that this context limited their scope for onward referral, as discussed in one focus group.

Celia yes, in [Kesforth] area, there’s not much at all. So, I’ve got a lot of clients, like even just social groups, so like crafty groups, things like that, there’s nothing in that [Kesforth] area. And if you are someone who struggles with anxiety and you can’t go too far from your home…

Dan They should go to [Jaytown], [Gatside]

Celia It’s still far. It’s a still long way to go if you can’t get out.

(Focus Group)

In the same focus group, Amy continued:

I totally agree. I mean part of my frustration is the fact that on my way home I drive through [Jaytown]. It’s clean. It’s tidy [laughter]. It’s a completely different type of area. And I find that, sort of structural poverty that is over here, it shouldn’t be like that. You know, you wouldn’t dream of the back alleys in [Jaytown] looking like that. It just wouldn’t happen. And I just found, “Well, why wouldn’t it happen? What is the mentality of the council or the this or the that that is allowing this area to be in the poverty?” And that for me, is a big part of why I don’t enjoy the job, because I just get frustrated with why our clients are in the position that they’re in.

(Amy_Focus Group)

Here, Amy highlights the way clients engaged with the intervention from disadvantaged positions and identifies a lack of local investment. In another encounter, a client described the closure of the local swimming pool as a barrier to him exercising, with transport to activities further afield often being prohibitive. Area level differences in provision were not the only constraining factors when it came to providing clients with options. There were recurrent concerns expressed about the capacity of the third sector to cope with an influx of referrals through social prescribing. Without adequate investment in the local voluntary and community sector, it was feared that their scope would be limited. One link worker shared a document that likened the situation to having ‘lots of travel agents and no holiday’ (Connected Voice, 2020). Overall, there were limited opportunities for link workers to adapt the role to navigate the impact of austerity, high levels of local deprivation and dwindling third sector capacity (Clayton et al., 2016).

The tensions between promoting individual choice and attending to collective conditions are well‐established. Through the sum of enhanced individual lifestyle choices, the intervention aimed to improve collective health, but individual choice was constrained by the conditions in which the collective lived (Mol, 2008. pp. 79–82). Link workers had limited capacity to reverse the impact of austerity and local funding cuts, putting the lived reality of link working at odds with discourses that promote the capacity of social prescribing to address the social determinants of health (Calderón‐Larrañaga et al., 2022).

## DISCUSSION

Our analysis demonstrates how the opposing logics of choice and care were presented and interacted within this intervention. Whilst the original aims of the intervention created the potential for care, over time, the structure and context of the intervention appeared to have aligned more with a logic of choice. Individual link worker practices were diverse, drawing differentially on both choice and care. Link worker subjectivities and organisational priorities were variably aligned, and link workers could sometimes feel frustrated that the practices they favoured, and that privileged care, were becoming increasingly difficult to deliver as the organisational priorities evolved.

### The organisational context

Complex funding arrangements meant that link workers’ capacity to exercise their own agency was increasingly constrained as they came under increasing pressure to meet targets and secure payment through the Social Impact Bond (SIB) model. SIBs sit within a wider agenda of New Public Management, often focussed on outcomes. They are embedded in a logic of choice that also shapes interpretations of personalisation in health care (Joy & Shields, 2013; Needham, 2014). We identified tensions, also described elsewhere, between addressing the requirements of the SIB and conducting the care practices needed to attend to the local context (Lowe et al., 2019). In this instance, these tensions were intensified by the differential engagement of primary care, which supports previous findings that a more holistic, relational type of link working requires increasing levels of collaboration, with ‘increasingly equitable sharing of roles, responsibilities, rights and rewards’ (Kimberlee, 2015; Southby & Gamsu, 2018, p. e362). The impact of this context on delivery is perhaps unsurprising. Community health workers in the United States, for instance, have previously described how the ‘agenda less’ relationships they built with clients were undermined by embedded neoliberal health‐care structures and targets (Cain et al., 2021, pp. 353–385) that commonly align with a logic of choice (Mol, 2008).

### Power, choice and care

In addition to targets, some link workers also identified an increasing organisational focus on ‘activation’ and the promotion of behaviour change that could further limit their capacity to provide the undifferentiated support that aligned with a logic of care. Refracted through a governmentality lens, this approach to social prescribing created a new space with a widened gaze over individual conduct that could often focus on the *choice* to make lifestyle changes through the wielding of a new form of *pastoral power* (Foucault, 2008; Mattioni et al., 2021; Waring et al., 2016). Practices that promoted behaviour change aligned with social prescribing discourses of self‐management and independence (Calderón‐Larrañaga et al., 2022; Jones, 2018) and appeared, over time, to converge with the increasingly dominant logic of choice. Such practices also resonated with critiques of social prescribing that caution against *lifestyle drift* when presenting link working as a solution to structural inequality (Calderón‐Larrañaga et al., 2022; Gibson et al., 2021; Williams & Fullagar, 2019). However, it was not just those accessing link working who were the subjects of power. We also saw how link workers, and link working practices, were themselves shaped within different networks of power and power hierarchies within the wider organisation and primary care (Waring et al., 2016). When UK community pharmacists began offering routine health checks, they too became new conduits of power whilst also being themselves subject to disciplinary power (Atkin et al., 2021; Waring et al., 2016). Performing the checks gave them a new *pastoral gaze* to inspect individual conduct, but the pressure to meet targets subjected them to an *organisational gaze* that led to a dissonance between professional ideals and practical applications. Furthermore, introducing new ‘superficial sites of practice’ ensured a ‘more obvious governmentality (and the associated fragility of underfunded primary care and public health services) [was] concealed and protected’ (Atkin et al., 2021, p. 348). Whilst social prescribing discourses often claim to address the social determinants of health, we saw that front‐line link workers were not positioned to reverse sustained local socioeconomic deprivation and were constrained by an ongoing lack of investment in the local community.

Situating link workers within these dynamic power networks helps us understand how their routines were increasingly shaped by a logic of choice. However, we also saw link working practices that drew on a logic of care. For example, link workers paid impromptu visits to clients or texted them in‐between appointments, in practices that were sensitive to context and attended to clients’ circumstances. Whilst such examples could be framed simply as resistance to the shaping forces that drive and sustain a logic of choice (Jones, 2018; Waring et al., 2016), doing so risks overlooking the coexistence of the logics of choice and care. Indeed, the interaction observed between choice and care appeared to draw together different ways of understanding and practising social prescribing within this single intervention, such that even individual link worker practices were not fixed.

Whilst a governmentality perspective draws attention to the importance of power and the dominance of choice, through identifying care practices, we are careful not to dismiss link working as a neoliberal disciplining strategy. Instead, we perceive front‐line social prescribing more as an assemblage of practices and knowledge embedded in wider discourses and policy contexts. We build on the insights arrived at through a governmentality lens by drawing attention to the multiplicity inherent in front‐line interpretations of social prescribing. Through tracing the interacting logics of choice and care, we saw how this multiplicity generated a range of understandings, experiences and practices, which were enacted and resisted in different ways (Mol, 2002, 2008; Singleton, 2005; Will, 2017). Despite the gradual dominance of a logic of choice, link working retained a plasticity that might yet be harnessed in challenging such an approach and in meeting the variable needs of different groups (Husk et al., 2020). However, it remains possible that, over time, the increasing dominance of choice in wider policy and discourse could marginalise care, favour individualist approaches and ignore local context, concealing the need for wider investment, in much the same way that critics of personalisation have described elsewhere (Barnes, 2011; Needham, 2014; Needham & Glasby, 2014).

### Looking to the future

From the vantage point of some imagined future, Walker et al. (2017) have suggested that the arrival of social prescribing allowed GPs to refer ‘immiserated people’ to community services in a model favoured by neoliberal governments as a way to promote ‘patient choice’ and ‘cut state medical costs’. (2017, p. 7) All of this whilst a ‘besieged community sector’ waited to be recognised for the work they were already doing (Walker et al., 2017, p. 7). This would certainly resonate with some of our findings. Yet, looking back from a fictional 2050, Walker et al. also suggested social prescribing was a turning point, where community became central to helping people deal with ‘embodied suffering’ and the ‘understandable responses to life histories’ (Walker et al., 2017, p. 8). We propose that this vision of social prescribing would need to privilege care, resonating with recent calls for a ‘care‐based’ framing (Calderón‐Larrañaga et al., 2022). In this context, personalisation would become relational, moving the focus from choice to delivering care that foregrounded individual context and meaning, problematising outcomes‐based approaches to funding, evidence and future research.

We saw link workers negotiating context to help clients set their own goals, such as attending future appointments, through a logic of care that did little to satisfy the population level targets and outcomes of the intervention that were rooted in a logic of choice. Needham (2011, 2014) reminds us of the paradoxes and ambiguities in evidencing and delivering care within a personalisation agenda that privileges choice. In contrast to a logic of choice, a focus on care minimises targets and problematises the requirements of an evidence base, resonating with wider calls to overcome the ‘tyranny of what can be measured’ in health care (Heath, 2015; Mol, 2008; Povar, 1995, p. JS67).

To our knowledge, this is the first in‐depth ethnographic account of front‐line social prescribing. The chosen methodology allowed us to gain a rich insight into link working routines and trace emerging tensions in front‐line delivery through engaging with complexity.

The main limitations arise from the fieldwork being limited to one social prescribing intervention, making it difficult to generalise. However, our findings allow us to engage with new ways of thinking about social prescribing.

## CONCLUSION

Understanding how link working can become more relational through privileging care attends to debates on what approaches to deliver ‘for whom, and in what circumstances’ (Husk et al., 2020). Our findings could be relevant as newly formed Primary Care Networks and social prescribing collaboratives make choices about local funding arrangements, models of delivery, output metrics, evidence and targets. Through paying attention to care, there is an opportunity to overcome some of the constraints a logic of choice can place on link working, favouring models that deliver holistic and relational experiences of personalisation over those that responsibilise individuals. Furthermore, recognising how the powerful governmentality that can shape personalisation through a logic of choice can obscure the need for wider investment might ensure that transformational policy aims and objectives do not diverge from front‐line experience.

## AUTHOR CONTRIBUTIONS

**Bethan Griffith**: Data curation (Equal); Formal analysis (Lead); Methodology (Equal); Writing—original draft (Lead); Writing—review & editing (Equal). **Tessa Pollard**: Conceptualisation (Equal); Formal analysis (Supporting); Funding acquisition (Equal); Methodology (Equal); Project administration (Supporting); Supervision (Equal); Writing—review & editing (Lead). **Kate Gibson**: Formal analysis (Supporting); Investigation (Supporting); Methodology (Supporting); Project administration (Supporting); Writing—review & editing (Equal). **Jayne Jeffries**: Data curation (Equal); Investigation (Lead); Methodology (Equal). **Suzanne Moffatt**: Conceptualisation (Equal); Formal analysis (Supporting); Funding acquisition (Equal); Methodology (Equal); Project administration (Lead); Supervision (Equal); Writing—review & editing (Equal).

## Data Availability

The data that support the findings of this study are available on request from the corresponding author. The data are not publicly available due to privacy or ethical restrictions.

## ENDNOTES

^1^
GP practices in the UK are now working together with community, mental health, social care, pharmacy, hospital and voluntary services in their local areas in groups of practices known as primary care networks (PCNs) (NHS England, 2022).

^2^
COPD or Asthma; Diabetes (Type 1 or Type 2); Heart Disease; Epilepsy; Osteoporosis; any of these with depression and/or anxiety.

^3^
NHS ethical approval was not required, as provider organisations did not employ NHS staff.

^4^
Personal Independence Payment (PIP) is a UK government benefit that can help with extra living costs for those with both a long‐term physical or mental health condition or disability and difficulty doing certain everyday tasks because of that condition (UK Government, 2022a, 2022b).

^5^
Employment and Support Allowance (ESA) is a government benefit for those who have a disability or health condition that affects how much they can work. ESA provides money to help with living costs for those unable to work and support to get back into work if possible (UK Government, 2022a, 2022b).
